# Incremental Value of Apical Longitudinal Strain in Predicting High-Risk Apical Aneurysms in Patients with Hypertrophic Cardiomyopathy

**DOI:** 10.3390/diagnostics16040575

**Published:** 2026-02-14

**Authors:** Xin Hu, Xueqing Cheng, Yuwei Bao, Jie Tian, Shiliang Liu, Yaqin Yang, Qi Xu, Bingyi Zhang, Youbin Deng, Yongping Lu, Yani Liu

**Affiliations:** 1 Department of Medical Ultrasound, Wuhan Forth Hospital, Wuhan 430033, China; xinhu1212@163.com (X.H.);; 2 Department of Medical Ultrasound, Tongji Hospital, Tongji Medical College, Huazhong University of Science and Technology, 1095 Jiefang Road, Wuhan 430030, China; 3 Department of Medical Ultrasound, The Affiliated Hospital of Yunnan University (The Second People’s Hospital of Yunnan Province, Yunnan Eye Hospital), Wuhua District, Kunming 650021, China

**Keywords:** hypertrophic cardiomyopathy, apical aneurysm, adverse cardiovascular events, strain parameters, 5-year SCD risk scores

## Abstract

**Background**: Apical aneurysms have long been considered a critical risk marker for poor clinical outcomes in hypertrophic cardiomyopathy (HCM) individuals. This study aims to identify apical features associated with adverse outcomes and explore their incremental predictive value beyond the traditional sudden cardiac death (SCD) risk score model. **Methods**: From December 2019 to November 2024, 2318 HCM patients were diagnosed at Tongji Hospital. Ultimately, 65 HCM patients with apical aneurysms were included in the analysis, each having undergone conventional and contrast echocardiography, as well as speckle tracking echocardiography (STE). **Results:** With a median follow-up of 26 months, composite events occurred in 25 (38%) patients, while none occurred in 40 (62%). Multivariate Cox regression revealed that abnormal apical longitudinal strain average (LS-avg) significantly increased composite event risk (HR: 1.23; 95% CI: 1.02–1.48). For patients with a 5-year SCD risk score < 4% or aneurysm diameter < 20 mm, survival differed significantly between apical LS-avg ≥ −6.6% and <−6.6% (*p* < 0.05). Correct reclassification was 10.8% (7/65) for reduced 5-year SCD risk scores and 15.4% (10/65) for smaller aneurysms. Incorporating apical LS-avg into 5-year SCD risk score or aneurysm diameter assessment improved risk assessment (NRI: 67.7% and 66.2% for adverse event prediction). A likelihood ratio test showed that apical LS-avg enhanced prognostic accuracy in patients, with lower 5-year SCD risk scores and smaller aneurysms (all *p* < 0.001). **Conclusions**: Apical LS-avg may be associated with an increased risk of adverse cardiovascular events in HCM individuals who had apical aneurysms. On the basis of the conventional 5-year SCD risk score and aneurysm size, apical LS-avg may have the potential to be used to individually identify the high-risk group of this patient cohort, particularly among those with a 5-year SCD risk score < 4% and an aneurysm diameter < 20 mm.

## 1. Introduction

Hypertrophic cardiomyopathy (HCM), an inherited cardiac disease, is estimated to have a prevalence of 0.2% to 0.5%, classifying it as a relatively common genetic myocardial disorder [1]. It is characterized by myocardial hypertrophy, particularly in the interventricular septum. The complex pathophysiological mechanisms and diverse phenotypic characteristics of HCM pose significant challenges to patient prognosis. Reported in 2% to 5% of HCM individuals, left ventricular (LV) apical aneurysms represent a rare phenotypic manifestation that has been linked to LV mid-obstruction or chamber obliteration [2,3]. Previous studies have revealed that apical aneurysms are often linked to a higher incidence of adverse cardiovascular diseases and mortality, encompassing sudden cardiac death (SCD), sustained ventricular arrhythmia/ventricular fibrillation (VT/VF), thromboembolism, and progression to the terminal stage of the disease [4,5,6,7,8]. However, recent research has revealed that some apical aneurysms may remain stable over the long term without significant changes [9]. Therefore, individualized detection of high-risk apical aneurysms plays a critical role in optimizing risk stratification and enabling targeted management strategies for affected individuals.

Advances in multimodal cardiovascular imaging techniques have not only enhanced the ability to identify apical aneurysms in HCM patients but also helped identify individuals at higher risk of experiencing adverse cardiac outcomes [5,10,11]. Previous studies have demonstrated that the formation of apical aneurysms, when combined with other hallmarks of advanced disease, such as extensive myocardial fibrosis, non-sustained ventricular arrhythmia (NSVT), and impaired LV systolic function, signifies a high-risk phenotypic profile, representing a critical stage of disease progression [6]. Moreover, a large single-center cohort study by Lee et al. in 2022, which included 160 HCM patients with apical aneurysms, suggested that apical aneurysms measuring ≥2 cm were associated with a poorer prognosis compared to smaller aneurysms [12]. Conversely, a 2017 study by Rowin et al. revealed that no consistent association existed between the size of aneurysms and adverse cardiac outcomes [5]. Although traditional echocardiography and cardiac magnetic resonance (CMR) techniques are valuable in assessing cardiac structure and function, individual variability and disease complexity necessitate the introduction of novel, clinically feasible parameters to further predict adverse cardiac outcomes in HCM patients combined with apical aneurysms.

In recent years, speckle tracking echocardiography (STE) has been increasingly employed for apical mechanical analysis [13,14,15]. Furthermore, mechanical factors are closely related to myocardial fibrosis assessed by late gadolinium enhancement (LGE) and the prognosis of HCM [13,16]. In the present study, we utilized STE to analyze the global and regional (including apical/middle/basal regions) strain parameters of the left ventricle in HCM patients combined with apical aneurysms. We aimed to explore the specific apical characteristics related to adverse events and their incremental predictive value beyond the traditional SCD risk score model.

## 2. Methods

### 2.1. Study Population

**Figure 1:**
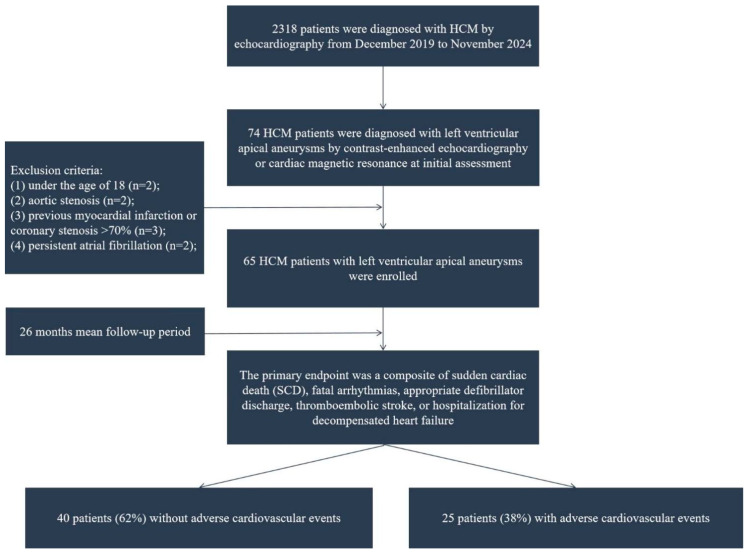
Patient flowchart.

This study has been approved by the Review Board of Tongji Hospital, and written consent has been obtained from all patients. This single-center study adhered to the principles of the Helsinki Declaration and was conducted in accordance with the protocol approved by the Ethics Committee of Tongji Medical College (approval number: 2022-S013, 2022-S013-1, 2022-S013-2, 2022-S013-3, 2022-S013-4, date: 23 February 2022; also registered at ClinicalTrials.gov, NCT05332691). Between December 2019 and November 2024, 2318 HCM patients were consecutively enrolled at Tongji Hospital. HCM was diagnosed according to guideline-recommended standardized criteria, defined as LV wall thickness ≥ 15 mm in adults, excluding cases explainable by pathological hemodynamic stress states [4,17]. Among them, 74 HCM patients (3.2%) were diagnosed with left ventricular (LV) apical aneurysm by contrast-enhanced echocardiography and/or cardiac magnetic resonance (CMR) at the initial assessment. Of these 74 patients, 69 underwent contrast-enhanced echocardiography, and 57 underwent CMR imaging. Apical aneurysm was characterized as a localized thin-walled segment in the LV apex, which has been confirmed by echocardiography and/or CMR to be independent of obstructive atherosclerotic coronary artery disease [9]. According to the exclusion criteria [18], patients under 18 years of age (*n* = 2), with aortic stenosis (*n* = 2), prior myocardial infarction or coronary stenosis > 70% (*n* = 3), or persistent atrial fibrillation (*n* = 2) were excluded. Ultimately, the study cohort comprised 65 HCM patients with confirmed apical aneurysms, as depicted in the enrollment flowchart (Figure 1).

### 2.2. Clinical Information

The medical history, laboratory data, electrocardiograms (ECGs), and CMR results of patients were all collected from electronic medical records. Non-sustained ventricular tachycardia (NSVT) was identified via 24 h Holter electrocardiography, defined as the onset of ≥3 ventricular ectopic beats in a row with a frequency of >100 beats per minute and a duration of <30 s [19]. The 5-year SCD risk score of the European Society of Cardiology was calculated in accordance with an externally validated and recognized 5-year SCD risk prediction model for HCM patients [20,21].

### 2.3. Transthoracic Echocardiography

Experienced echocardiographers performed transthoracic echocardiography (TTE) using a device specialized in cardiac imaging. The equipment used in this study, model GE Vivid E95, is manufactured by Vingmed Ultrasound in Horten, Norway. Following optimization of the examination parameters, multi-sectional views were obtained using the M5Sc transducers. All image acquisition and quantitative analysis were standardized in accordance with guideline requirements [22]. The size of apical aneurysm was characterized by the greatest transverse width from mid-systolic to end-systolic on echocardiography in the two-, three-, and four-chamber views [12]. An ultrasound contrast agent (SonoVue; Bracco) was used to accurately visualize the LV apex during the examination. LV apical/mid/basal obstruction was defined as a peak instantaneous pressure gradient ≥ 30 mmHg in the apical/mid/basal location at rest or during stress testing [2]. We measured the maximum wall thickness (MWT) of the LV apical/mid/basal region on short-axis images at end-diastole. We computed left ventricular ejection fraction (LVEF) and end-diastolic/systolic volume indices using the improved Simpson biplane method.

### 2.4. Speckle Tracking Echocardiography

We conducted strain analysis on the LV myocardium of the patients using speckle tracking echocardiography (STE) software—specifically, EchoPAC version 2.4 by GE Vingmed Ultrasound. By systematically choosing the three most compatible standard apical views and efficiently recognizing endocardial boundaries, the software ensured the tracking of myocardial motion. Endocardial boundaries were manually adjusted as necessary by the operator to ensure tracking accuracy. Post-analysis, the software programmatically generated a bull’s-eye diagram of the 18 LV segments, which visualized global longitudinal strain (GLS) and segmental longitudinal strain (LS) across cardiac segments. The calculation of GLS entailed averaging LS values from 18 segments, and the numerical value was automatically displayed. The left ventricle was conventionally divided into apical, middle, and basal regions. According to the LV 18-segment model, the regional strain parameters of the apical, middle, and basal regions were further derived by calculating the mean value of the segmental strain parameters of the corresponding regions.

### 2.5. Clinical Follow-Up

Adverse cardiovascular event data were collected via the hospital’s electronic medical record system and systematic telephone interviewing. We defined the primary clinical endpoint as a composite endpoint, encompassing SCD, sustained VT/VF, appropriate ICD discharges, new-onset thromboembolic stroke (confirmed by a consultation with a neurologist), or hospitalization for decompensated heart failure. The event-free survival period was defined as the period from the date of the first confirmed diagnosis until the occurrence of the primary endpoint event, or until the end of the study.

### 2.6. Statistical Analysis

Normally distributed variables were described as mean ± SD, with intergroup comparisons via Student’s *t*-test. Non-normal data were presented as median (interquartile range) and compared using the Mann–Whitney U test. Categorical data were expressed as frequencies (percentages), compared by chi-square or Fisher’s exact test. Multiple groups were analyzed via ANOVA. Variables with significant association (*p* < 0.05) with the composite endpoint were included in subsequent multivariate Cox regression. A restricted cubic spline (RCS) model characterized the non-linear relationship between the research parameter and the endpoint’s hazard ratio. Kaplan–Meier estimated survival probability, and a log-rank test assessed intergroup survival differences. Net reclassification improvement (NRI) evaluated risk category reclassification; an optimistic corrected C-index compared model predictive performance. All analyses used IBM SPSS 25.0 and R 4.4.3, with two-tailed *p* < 0.05 as the significance threshold.

## 3. Results

### 3.1. Baseline Clinical Features and Outcomes

This study enrolled a total of 65 patients with HCM who had apical aneurysms. The average age of these patients was 52.4 ± 11.8 years, and 80% of them were male. As shown in Table 1, the detailed baseline clinical characteristics and outcome data of the research subjects in each research group have been summarized. We found that 4 patients (6.1%) had a family history of HCM, 3 patients (4.3%) had a family history of SCD, and 34 patients (52.3%) were asymptomatic at baseline. Among all HCM patients with apical aneurysms, statistical analyses revealed that no significant differences were observed in general parameters (gender and age), body mass index, blood pressure, heart rate, bundle branch block, QRS duration, NT-proBNP, hs-cTnI, symptoms, atrial fibrillation, anticoagulation therapy at baseline, and NYHA functional class between two groups (event vs. non-event; *p* > 0.05). However, compared to the group without adverse cardiovascular events, the incidence of NSVT in the group with adverse cardiovascular events increased significantly (*p* < 0.05). Furthermore, compared to the group without any events, the 5-year SCD risk score was significantly higher in the event group (*p* < 0.05). There were 49 patients (75.3%) with a low-risk score (less than 4%), 10 patients (15.3%) with a moderate-risk score (ranging from 4% to 6%), and 6 patients (9.2%) with a high-risk score (greater than 6%). During the median follow-up period of 26 months, with a range spanning from 6 to 51 months, the composite events occurred in 25 patients (38.5%). Among them, 1 patient experienced SCD, 4 patients experienced sustained VT/VF or appropriate ICD discharges, 17 patients experienced new-onset thromboembolic stroke, and 9 patients were admitted to the hospital due to the deterioration of their heart failure conditions. Compared to the group without any events, anticoagulation therapy during the follow-up period was significantly higher in the event group (*p* < 0.05).

### 3.2. Baseline Imaging Parameters

**Figure 2:**
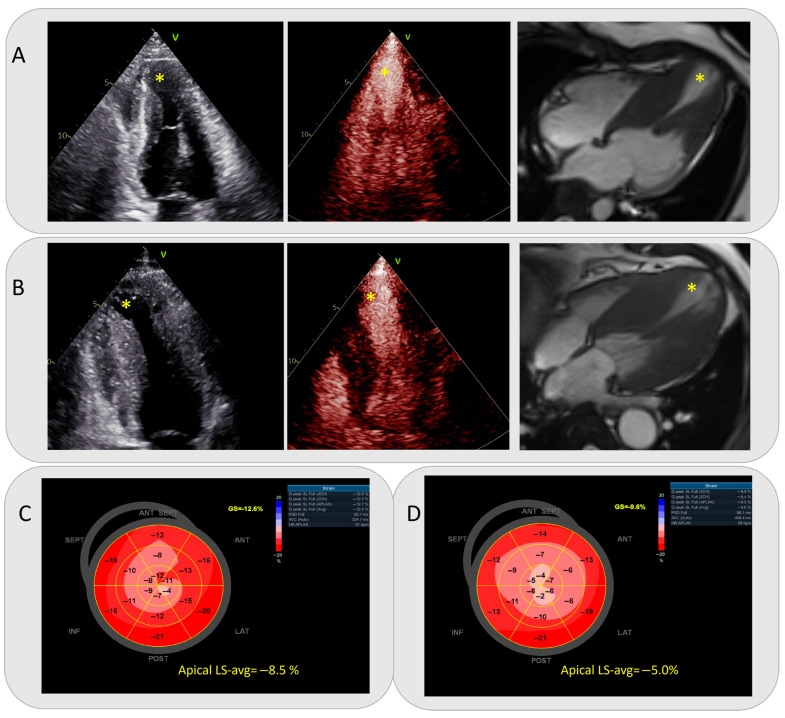
(**A**,**C**) A HCM patient with apical aneurysm did not experience any adverse cardiovascular events. A 20 mm apical aneurysm was revealed on two-dimensional transthoracic echocardiogram, contrast echocardiogram, and cardiac magnetic resonance, respectively. The patient’s 5-year SCD risk score was 3.0%, and apical LS-avg was −8.5% on a bull’s-eye map of speckle tracking imaging. The patient’s 5-year SCD risk score was 3.0%, and apical LS-avg was −8.5% on a bull’s- eye map of speckle tracking imaging. (**B**,**D**) Two-dimensional transthoracic echocardiogram, contrast echocardiogram, cardiac magnetic resonance image, and bull’s-eye map of speckle tracking imaging of an HCM patient with apical aneurysm who experienced adverse cardiovascular events. The patient had an apical aneurysm with a diameter of 23 mm, a 5-year SCD risk score of 3.4%, and an apical LS-avg value of −5.0%. The apical aneurysms on the images are marked with yellow asterisks. Abbreviations as in Table 1 and Table 2.

Table 2 presents study patients’ imaging data. Compared with non-adverse-event HCM patients with apical aneurysms, the event group had significantly larger total MWT, apical MWT, and apical aneurysm size (all *p* < 0.05). No significant differences were found in middle/basal MWT, intracavitary obstruction (location/extent), mid-systolic signal void/velocity drop, left atrial size, left ventricular volume indexes, or LVEF between the two groups. Analysis of 195 regional strain parameters (apical/middle/basal) showed that the event group had significantly abnormal global strain (GLS) and regional apical/middle LS-avg (all *p* < 0.05), but no abnormal basal LS-avg vs. the non-event group (*p* > 0.05). Typical cases of events that occurred and those that did not occur, including two-dimensional echocardiograms, contrast echocardiograms, CMR images, and a bull’s-eye map of STE, are shown in Figure 2.

### 3.3. Survival Analysis

The findings derived from univariate and multivariate Cox regression analyses of the composite primary events were meticulously summarized in Table 3. Univariable Cox regression analysis for the composite primary events identified the following statistically significant variables: NSVT (HR: 4.82; 95% CI: 1.96–11.84; *p* < 0.001), 5-year SCD risk score of European Society (HR: 1.24; 95% CI: 1.03–1.50; *p* = 0.021), MWT-Apical (HR: 1.12; 95% CI: 1.01–1.24; *p* = 0.035), size of aneurysm (HR: 1.09; 95% CI: 1.04–1.14; *p* < 0.001), LGE (HR: 7.85; 95% CI: 1.01–61.06 *p* = 0.049), GLS (HR: 1.19; 95% CI: 1.06–1.34; *p* = 0.004), apical LS-avg (HR: 1.26; 95% CI: 1.13–1.42; *p* < 0.001). All of the aforementioned variables with *p* < 0.05 and judged by senior echocardiography experts to have clinical significance were further included in the multivariate Cox regression analysis. As not all patients underwent LGE imaging, the LGE results were not included. In the final model, apical LS-avg remained statistically significant (HR: 1.23; 95% CI: 1.02–1.48; *p* = 0.029), indicating that apical LS-avg was associated with an increased risk of adverse cardiovascular events in HCM individuals who had apical aneurysms.

**Figure 3:**
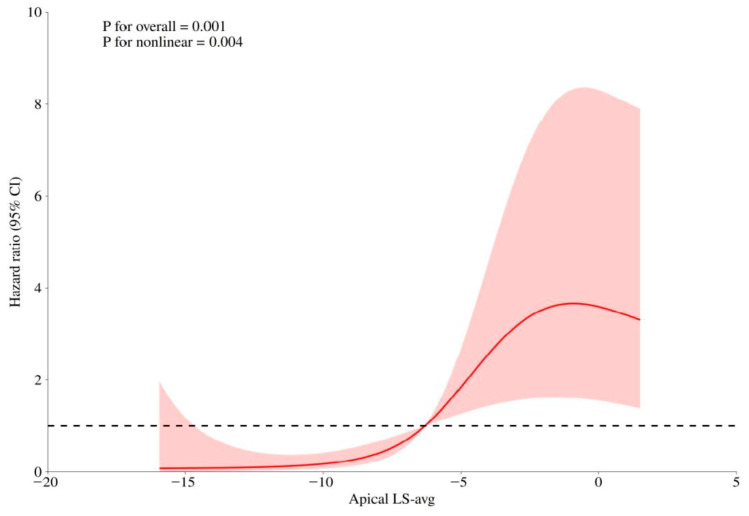
Restricted cubic splines of non-linear association between apical LS-avg and hazard ratio. The bold red lines indicate the pooled restricted cubic spline model, and the red-filled area indicates the 95% confidence intervals of the pooled curve. Abbreviations as in Table 2.

**Figure 4:**
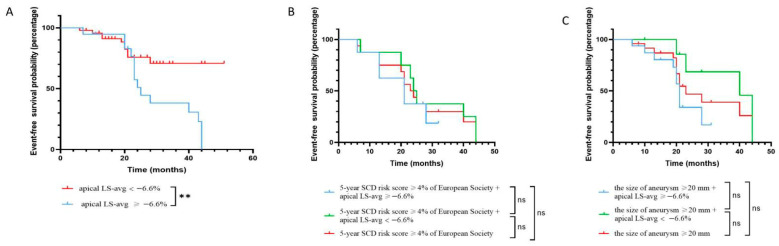
Event-free survival. (**A**) Kaplan–Meier survival curves of HCM patients with apical aneurysms stratified into two groups according to the average value of apical LS-avg. (**B**) Kaplan–Meier survival curves for adverse events prediction in patients with 5-year SCD risk score ≥ 4% by apical LS-avg. There was no significant difference in event-free survival rate between higher 5-year SCD risk score groups with apical LS-avg < −6.6% or ≥−6.6%. (**C**) Kaplan–Meier survival curves for adverse events prediction in patients with the size of aneurysm ≥ 20 mm by apical LS-avg. There was no significant difference in event-free survival rate between higher 5-year SCD risk score groups with apical LS-avg < −6.6% or ≥−6.6%. Abbreviations as in Table 1 and Table 2. ** *p* < 0.01.

Using restricted cubic spline curves, the continuous association between apical LS-avg and the risk of adverse outcomes was assessed based on the Cox regression model. As depicted in Figure 3, the elevation of abnormal apical LS-avg was significantly related with an increased risk of adverse outcomes (*p* < 0.01 for the non-linear relationship). As illustrated in Figure 4A, compared with patients whose apical LS-avg < −6.6%, the event-free survival rates in individuals with apical LS-avg ≥ −6.6%, according to the average value of apical LS-avg, was significantly lower (log-rank *p* < 0.05).

### 3.4. Incremental Value of Apical Longitudinal Strain

**Figure 5:**
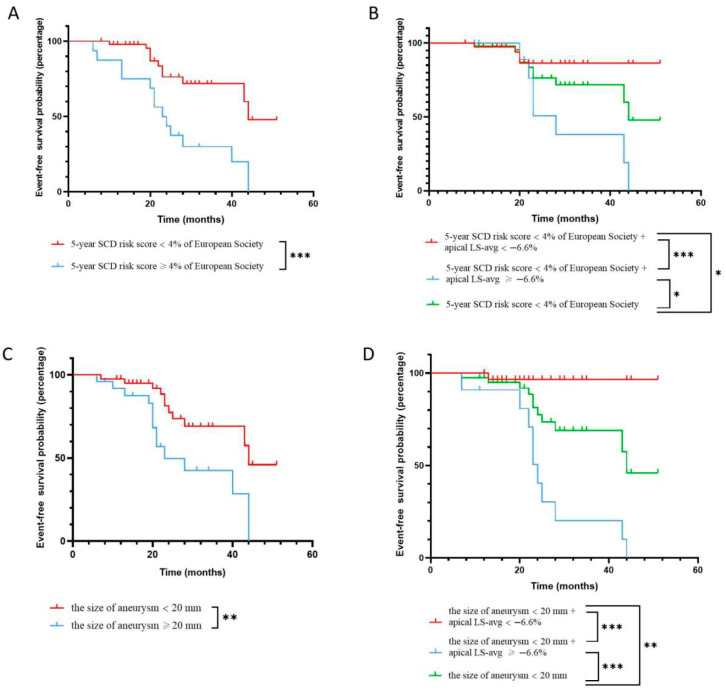
Event-free survival. (**A**) Kaplan–Meier survival curves of HCM patients with apical aneurysms stratified into two groups according to 5-year SCD risk score ≥ 4%. The event-free survival rate of patients with a 5-year SCD risk score ≥ 4% was significantly reduced. (**B**) Kaplan–Meier survival curves for adverse events prediction in patients with 5-year SCD risk score < 4% by apical LS-avg. The lower 5-year SCD risk score groups with apical LS-avg < −6.6% or ≥−6.6% showed significant event-free survival rate differences compared to the lower 5-year SCD risk score group alone. (**C**) Kaplan–Meier survival curves of HCM patients with apical aneurysms stratified into two groups according to the size of aneurysm ≥ 20 mm. (**D**) Kaplan–Meier survival curves for adverse events prediction in patients with the size of aneurysm < 20 mm by apical LS-avg. The smaller aneurysm groups with apical LS-avg < −6.6% or ≥−6.6% showed significant event-free survival rate differences compared to the smaller aneurysm group alone. Abbreviations as in Table 1 and Table 2. * *p* < 0.05, ** *p* < 0.01, and *** *p* < 0.001.

Among individuals who had 5-year SCD risk scores of <4% or ≥4 and an aneurysm size of <20 mm or ≥20 mm, the statistical difference in event-free survival rates was significant, as depicted in Figure 5A,C. Innovatively, we combined the value of apical LS-avg (≥−6.6% or <−6.6%) respectively with the 5-year SCD risk score (<4% or ≥4%) and aneurysm size (<20 mm or ≥20 mm) to evaluate the incremental predictive value of the apical LS-avg in improving the stratification of adverse cardiovascular events over the traditional SCD risk score model. For individuals who had a 5-year SCD risk score ≥ 4, no significant difference in event-free survival rate between higher 5-year SCD risk score groups who had apical LS-avg < −6.6% and ≥−6.6% was found (Figure 4B). Similarly, in the larger aneurysm group, there was no significant difference in event-free survival rates between the group with apical LS-avg < −6.6% and ≥−6.6% (Figure 4C).

**Figure 6:**
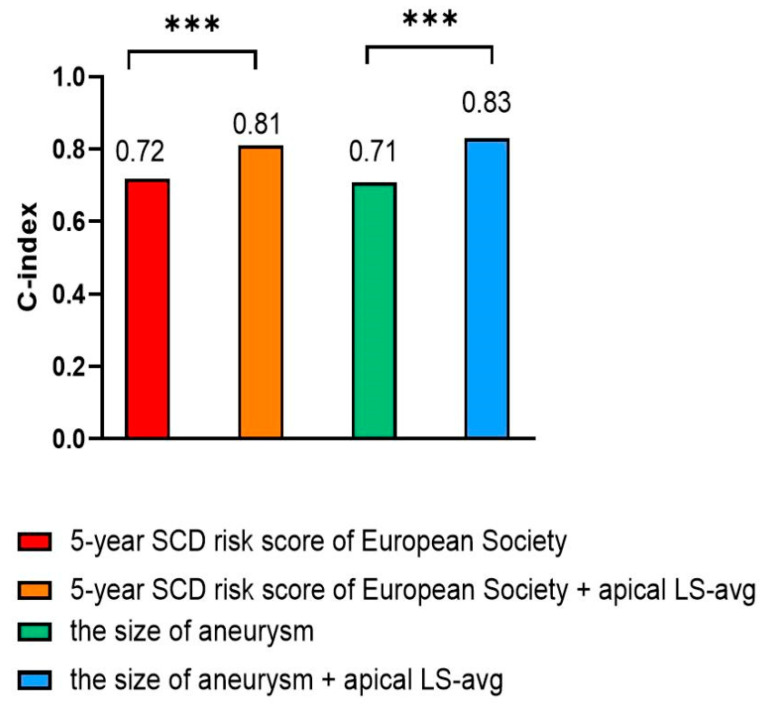
Model performance was evaluated using the C-index, and model comparison has been performed using the likelihood-ratio test for nested models. Abbreviations as in Table 1 and Table 2. *** *p* < 0.001.

Specifically, for patients who had a 5-year SCD risk score < 4% or an aneurysm size < 20 mm, a statistical difference in event-free survival rates was found between those with apical LS-avg ≥ −6.6% and those with apical LS-avg < −6.6% (log-rank *p* < 0.05) (Figure 5B,D). When apical LS-avg ≥ −6.6%, event-free survival rates significantly decreased in those who had a 5-year SCD risk score < 4% or an aneurysm size < 20 mm. In HCM patients complicated by apical aneurysms, significant stratification of adverse cardiovascular events was observed among those with a lower 5-year SCD risk score or a smaller aneurysm size. This indicates that incorporating apical LS-avg into the 5-year SCD risk score and considering aneurysm size contributes to significantly improving the risk stratification of poor outcomes in HCM patients who had apical aneurysms. Correct reclassification was achieved for 10.8% (7 of 65) of individuals with lower 5-year SCD risk scores and 15.4% (10 of 65) of patients with smaller aneurysms (Table 4). Incorporating apical LS-avg into the 5-year SCD risk score or aneurysm size significantly improved risk assessment, resulting in an NRI of 67.7% and 66.2% in the prediction of adverse cardiovascular events, respectively (Table 4). Accordingly, incorporating apical LS-avg into the 5-year SCD risk score or aneurysm size significantly improved the predictive performance, with C-index values of 0.81 and 0.83, respectively (Figure 6). The likelihood ratio test demonstrated that apical LS-avg significantly enhanced the prognostic accuracy of poor outcomes in individuals who had lower 5-year SCD risk scores and smaller aneurysms. This result highlights the significance of apical LS-avg with risk scores and the size of aneurysm for refined risk stratification.

### 3.5. Variability Analysis

Bland–Altman analysis verified that apical/mid/basal LS-avg showed excellent inter- and intra-observer reproducibility, with relevant results detailed in Supplementary Table S1.

## 4. Discussion

Apical aneurysms in HCM patients have long been considered to be associated with poor outcomes, including sustained VT/VF, thromboembolism, and SCD [4,12]. The 2024 AHA/ACC/AMSSM/HRS/PACES/SCMR guidelines for HCM state that although the progressive mechanisms underlying high-risk morphological features—including apical aneurysm, extensive LGE, and systolic dysfunction—remain unclear, these features are of critical importance in clinical management, and longitudinal assessment with CMR imaging may yield substantial reference value [1]. Furthermore, the 2023 ESC guidelines recommended that individualized ICD decisions should be based on clear risk factors, rather than solely on the presence of an apical aneurysm [17]. Therefore, the prediction and prevention of severe adverse outcomes for those patients represent an urgent clinical priority, while in-depth analysis of the apical aneurysms’ characteristics and exploration of novel risk markers assume critical significance.

It is reported that when the formation of the apical aneurysm is associated with other important features, it represents an advanced stage in the progression of the disease [6]. These high-risk features include extensive myocardial scarring, NSVT, and impaired LV systolic function. O’Mahony et al. reported that among patients with 5-year SCD risk scores of ≥4%, for every 16 cases of ICD surgeries performed, there was a possibility that 1 patient would avoid the occurrence of SCD within 5 years [21]. The subtypes of such apical aneurysms in HCM patients are emphasized in the 2024 AHA/ACC/AMSSM/HRS/PACES/SCMR guidelines for HCM [1]. In this study, we aim to explore the specific apical characteristics associated with adverse events and to investigate their incremental predictive value beyond the traditional SCD risk score model. Furthermore, the present study thoroughly investigates the risk of poor outcomes in HCM individuals complicated by apical aneurysms, specifically those with lower 5-year SCD risk scores and smaller aneurysms.

Previous studies have shown that abnormal strain values observed in HCM patients are closely associated with poor prognosis and have been included in the clinical practice management guidelines for HCM [1,15,23]. Moreover, strain has been extensively applied in apical mechanical analysis [13,14,15]. This study aims to explore the potential relationship between apical LS-avg and adverse cardiac events in patients with HCM combined with apical aneurysms. In our study, about 38% of the HCM patients with apical aneurysms experienced composite events. Additionally, patients who reached the endpoints exhibited significantly more severe impairment of apical LS-avg compared to those without endpoint events. In addition, the event-free survival rate of patients with severe abnormalities in the apical LS-avg was significantly lower than that of patients with mild abnormalities in the apical LS-avg. The abnormality of apical LS-avg value may be associated with potential myocardial fibrosis and/or myocardial ischemia in apical aneurysm. Given the small sample size of only 25 positive cases in this study, the constructed multivariate predictive model incorporated a relatively large set of variables. With a cautious interpretive stance, we propose that apical LS-avg may be associated with an increased risk of adverse cardiovascular events in HCM individuals who had apical aneurysms. It is worth noting that this research is exploratory in nature and may not be universally applicable.

More importantly, this study further analyzed strain characteristics of patients with 5-year SCD risk scores < 4% or aneurysm size < 20 mm. In 2022, Lee et al. (with a large single-center cohort) found that HCM apical aneurysm size negatively correlated with prognosis, suggesting that a size ≥ 20 mm warrants prophylactic anticoagulation and ICD primary prevention [12], though smaller aneurysms do not rule out thromboembolic events. Controversially, Rowin et al. argued that aneurysm size is unrelated to HCM adverse events, noting that smaller aneurysms may still be a structural basis for thrombosis [5]. Lorenzini et al. proposed that ICD implantation should prioritize secondary prevention and target patients with aneurysms sufficiently large to cause reduced LVEF [6,9]. Moreover, it was emphasized that ICD decisions should not depend solely on LV apical aneurysm presence, making risk assessment/stratified management critical for HCM patients with small apical aneurysms (at risk of under-recognition). Thus, for patients with these smaller aneurysms and a lower 5-year SCD risk score, it is particularly important to identify their high-risk factors. Our analysis shows that when patients with lower 5-year SCD risk scores or smaller aneurysms are subdivided by apical LS-avg (<−6.6% vs. ≥−6.6%), event-free survival rates differ significantly vs. classification solely by low risk score or small aneurysm. Considering that there are a large number of cases of new thromboembolic events, perhaps we should improve the management of such patients.

Currently, there is no specific risk scoring model for the HCM subtype with apical aneurysms. Based on the correlation between abnormal longitudinal strain average and poor cardiovascular outcomes in these patients, this study developed a tailored risk scoring model. For patients with lower 5-year SCD risk scores, integrating apical LS-avg with the score enabled correct reclassification of 10.8% (NRI = 67.7%). For those with smaller aneurysms, 15.4% achieved correct reclassification (NRI = 66.2%). This indicates that adding apical LS-avg to 5-year SCD risk score and aneurysm size significantly improves risk stratification, especially for patients with lower SCD risk scores or smaller aneurysms—thus, accurately assessing the apical LS-avg may contribute to optimizing prophylactic anticoagulation and ICD implantation strategies. Nevertheless, when more embolism results are combined with the risk score for cardiogenic sudden death, it may lead to an overestimation of the results.

### Limitations

This study had the following limitations: (1) Findings came from a single-center, small subset of HCM patients with apical aneurysms. (2) There was a lack of 3D speckle tracking echocardiography to reflect multi-dimensional, stereoscopic tissue lesion features in these patients. (3) Only 46 patients had available CMR–LGE results, which may have underestimated the predictive value of the LGE indicator and precluded its inclusion in the multivariate analysis. (4) Owing to the fact that only 25 positive events were recorded in this study, the number of variables included in the multivariate prediction model was relatively excessive. Thus, a prospective study with more such HCM patients and long-term observation is needed to verify apical longitudinal strain average’s value in predicting poor cardiovascular outcomes.

## 5. Conclusions

Apical LS-avg may be associated with an increased risk of adverse cardiovascular events in HCM individuals who had apical aneurysms. On the basis of the conventional 5-year SCD risk score and aneurysm size, apical LS-avg may have the potential to be used to identify the high-risk group within this patient cohort, particularly among those with a 5-year SCD risk score < 4% and an aneurysm diameter < 20 mm. It is worth noting that this research is exploratory in nature and may not be universally applicable.

## Figures and Tables

**Table 1:** Baseline characteristics and outcomes in HCM patients with LV apical aneurysm.

	All (*n* = 65)	Without Adverse Cardiovascular Events (*n* = 40)	With Adverse Cardiovascular Events (*n* = 25)	*p* Value
Age, years	52.4 ± 11.8	51.5 ± 12.5	53.6 ± 10.6	0.478
Male, *n* (%)	52 (80.0)	30 (75.0)	22 (88.0)	0.610
Body mass index, kg/m^2^	23.4 (1.7, 25.7)	22.6 (1.7, 25.7)	24.4 (1.7, 25.7)	0.438
Systolic blood pressure, mmHg	118.0 (82.0, 133.0)	112.0 (84.2, 133.2)	129.0 (78.0, 133.0)	0.613
Diastolic blood pressure, mmHg	73.0 (29.0, 81.0)	73.0 (30.1, 81.0)	76.0 (26.9, 81.0)	0.603
Heart rate, beats/min	79.0 (68.0, 101.0)	79.0 (68.0, 104.7)	81.0 (69.0, 93.0)	0.887
BBB, *n* (%)	15 (23.4)	10 (25.6)	5 (20.0)	0.921
QRS duration, msec	97.2 ± 30.8	97.2 ± 31.5	97.1 ± 30.7	0.995
NSVT (24-h holter), *n* (%)	17 (26.1)	1 (2.5)	16 (64.0)	**<0.001**
NT-proBNP, pg/mL	946.8 (442.5, 2052.0)	1045.0 (347.5, 1970.5)	916.4 (529.0, 2137.0)	0.675
hs-cTnI, pg/mL	36.1 (16.0, 218.5)	28.0 (16.0, 120.4)	93.8 (23.0, 232.0)	0.266
Chest pain, *n* (%)	29 (44.6)	19 (47.5)	10 (40.0)	0.743
Dyspnea, *n* (%)	8 (12.3)	4 (10.0)	4 (16.0)	0.060
Hypertension, *n* (%)	20 (31.7)	10 (25.6)	10 (41.6)	0.654
Diabetes, *n* (%)	8 (12.3)	6 (15.0)	2 (8.0)	0.554
Atrial fibrillation	2(3.1)	2 (4.8)	0 (0.0)	0.283
Unexplained syncope, *n* (%)	8 (12.3)	2 (5.0)	6 (24.0)	0.529
NYHA functional class, *n* (%)				
I	34 (52.3)	19 (47.5)	15 (60.0)	0.326
II	19 (29.2)	13 (32.5)	6 (24.0)	0.464
III	9 (13.9)	5 (12.5)	4 (16.0)	0.977
IV	3 (4.6)	3 (7.5)	0 (0.0)	0.427
Disease-causing mutation, *n* (%)	9 (16.6)	7 (20.0)	2 (10.5)	0.185
Coronary heart disease, *n* (%)	8 (12.3)	2 (5.0)	6 (24.0)	0.308
Family history of HCM, *n* (%)	4 (6.1)	1 (2.5)	3 (12.0)	0.674
Family history of SCD, *n* (%)	3 (4.6)	1 (2.5)	2 (8.0)	0.499
Anticoagulant therapy at baseline	3 (4.7)	2 (4.9)	1(4.5)	0.954
Anticoagulant therapy during follow-up	26 (40.0)	6(15.0)	20(80.0)	**<0.001**
5-year SCD risk score of European Society, %	2.9 ± 1.6	2.2 ± 0.9	4.1 ± 1.9	**<0.001**
5-year SCD risk score categories of European Society				
<4%, *n* (%)	49 (75.3)	38 (95.0)	11 (44.0)	**<0.001**
4–6%, *n* (%)	10 (15.3)	2 (5.0)	8 (32.0)	**<0.001**
>6%, *n* (%)	6 (9.2)	0 (0.0)	6 (24.0)	**<0.001**
Adverse cardiovascular events				
SCD, *n* (%)	1 (1.5)	0 (0.0)	1 (4.0)	0.385
Sustained VT/VF or appropriate ICD therapy, *n* (%)	4 (6.2)	0 (0.0)	4 (16.0)	**0.037**
New-onset thromboembolic stroke, *n* (%)	17 (26.2)	0 (0.0)	17 (68.0)	**<0.001**
Hospitalization due to worsening heart failure, *n* (%)	9 (13.9)	0 (0.0)	9 (36.0)	**<0.001**

NSVT, Non-sustained ventricular tachycardia; BBB, bundle branch block; NT-proBNP, N-terminal pro-brain natriuretic peptide; hs-cTnI, high-sensitivity cardiac troponin I; NYHA, New York Heart Association; HCM, hypertrophic cardiomyopathy; SCD, sudden cardiac death. Data are presented as mean ± SD or median (interquartile range) for continuous variables, and count (%) for categorical variables. Statistically significant comparisons (*p* < 0.05) are displayed in bold.

**Table 2:** Baseline imaging characteristics in HCM patients with LV apical aneurysm.

	All (*n* = 65)	Without Adverse Cardiovascular Events (*n* = 40)	With Adverse Cardiovascular Events (*n* = 25)	*p* Value
Conventional echocardiographic parameters				
MWT-Total, mm	21.3 ± 5.4	19.9 ± 4.1	23.4 ± 6.4	**0.019**
MWT-Apical, mm	15.9 ± 4.9	14.9 ± 4.3	17.6 ± 5.6	**0.028**
MWT-Mid, mm	18.8 ± 6.1	17.9 ± 5.1	20.1 ± 7.4	0.196
MWT-Basal, mm	17.0 ± 5.9	16.8 ± 5.1	17.3 ± 7.2	0.742
LA diameter, mm	40.5 ± 6.2	40.2 ± 6.4	41.1 ± 5.9	0.577
Mid-systolic signal void, *n* (%)	23 (35.4)	14 (35.0)	9 (36.0)	0.935
Mid-systolic drop in velocities, *n* (%)	23 (35.4)	14 (35.0)	9 (36.0)	0.474
Obstructive HCM, *n* (%)				
Apical	32 (49.2)	17 (42.5)	15 (60.0)	0.455
Mid	30 (46.2)	17 (42.5)	13 (52.0)	0.325
Basal	23 (35.4)	16 (40.0)	7 (28.0)	0.935
Apical HCM, *n* (%)	38 (58.462)	22 (55.0)	16 (64.0)	0.202
Maximum pressure gradient, mmHg	41.5 (10.0, 67.5)	36.50 (10.0, 64.3)	50.5 (12.7, 70.0)	0.418
E/A	0.8 (0.6, 1.2)	0.8 (0.6, 1.1)	0.8 (0.7, 1.3)	0.956
E/e’	14.87 ± 5.73	14.93 ± 6.12	14.79 ± 5.19	0.929
LVEF, %	63.2 ± 9.1	64.4 ± 7.4	61.2 ± 11.2	0.161
Thrombus, *n* (%)	3 (4.62)	1 (2.5)	2 (8.00)	0.674
Size of aneurysm, mm	17.6 ± 8.1	15.0 ± 6.7	21.5 ± 8.8	**<0.001**
Size of aneurysm < 20 mm, *n* (%)	41 (63)	30 (75)	11 (44)	0.111
Size of aneurysm ≥ 20 mm, *n* (%)	24 (37)	10 (25)	14 (56)	**<0.001**
LV end-diastolic volume index (mL/m^2^)	51.0 ± 11.7	50.4 ± 11.3	52.0 ± 12.4	0.646
LV end-systolic volume index (mL/m^2^)	20.1 ± 7.3	20.3 ± 7.9	19.6 ± 6.1	0.712
Cardiac magnetic resonance				
Late gadolinium enhancement, *n* (%)	40 (87.0)	20 (80.0)	20 (95.2)	0.276
Strain parameter				
GLS, %	−9.7 ± 3.2	−11.1 ± 2.8	−7.7 ± 2.8	**<0.001**
Apical LS-avg, %	−6.6 ± 5.9	−9.8 ± 4.2	−1.6 ± 4.7	**<0.001**
Mid LS-avg, %	−7.7 ± 4.4	−9.1 ± 4.4	−5.5 ± 3.6	**<0.001**
Basal LS-avg, %	−12.5 ± 4.6	−13.3 ± 4.0	−11.2 ± 5.1	0.074

MWT, maximum wall thickness; LA, left atrial; HCM, hypertrophic cardiomyopathy; E/A, mitral inflow peak early velocity/mitral inflow peak late velocity; E/e′, mitral inflow peak early velocity/mitral annular peak early velocity; LVEF, left ventricular ejection fraction; GLS, global longitudinal strain; LS, longitudinal strain. Data are presented as mean ± SD or median (interquartile range) for continuous variables, and count (%) for categorical variables. Statistically significant comparisons (*p* < 0.05) are displayed in bold.

**Table 3:** Univariable and multivariate Cox regression analysis of composite primary events.

	Univariable Analysis		Multivariate Analysis	
	HR (95% CI)	*p* Value	HR (95% CI)	*p* Value
Age, years	1.04 (0.98–1.09)	0.192		
Male, *n* (%)	2.21 (0.51–9.54)	0.289		
Hypertension, *n* (%)	0.88 (0.34–2.29)	0.788		
Diabetes, *n* (%)	0.89 (0.20–3.94)	0.876		
Family history of SCD, *n* (%)	0.79 (0.18–3.44)	0.752		
Family history of HCM, *n* (%)	2.71 (0.77–9.58)	0.121		
NSVT, *n* (%)	4.82 (1.96–11.84)	**<0.001**	2.57 (0.94–7.02)	0.066
MWT-Total, mm	1.06 (0.99–1.13)	0.101		
MWT-Apical, mm	1.12 (1.01–1.24)	**0.035**	0.99 (0.88–1.12)	0.928
LA diameter, mm	1.01 (0.94–1.08)	0.832		
Mid-systolic signal void, *n* (%)	1.57 (0.62–3.97)	0.344		
Mid-systolic drop in velocities, *n* (%)	0.97 (0.89–1.05)	0.422		
Maximum pressure gradient, mmHg	0.96 (0.38–2.48)	0.941		
E/A	0.64 (0.19–2.14)	0.469		
E/e’	0.99 (0.91–1.09)	0.894		
Size of aneurysm, mm	1.09 (1.04–1.14)	**<0.001**	1.01 (0.94–1.08)	0.814
LV end-diastolic volume index (mL/m^2^)	0.99 (0.96–1.03)	0.675		
LV end-systolic volume index (mL/m^2^)	0.97 (0.89–1.05)	0.422		
Late gadolinium enhancement, *n* (%)	7.85 (1.01–61.06)	**0.049**		
LVEF, %	0.97 (0.93–1.01)	0.13		
GLS, %	1.19 (1.06–1.34)	**0.004**	1.02 (0.85–1.21)	0.855
Apical LS-avg, %	1.26 (1.13–1.42)	**<0.001**	1.23 (1.02–1.48)	**0.029**
Mid LS-avg, %	1.07 (1.00–1.14)	0.056		
Basal LS-avg, %	1.04 (0.96–1.13)	0.331		

HR, hazard ratio; CI, confidence interval. Other abbreviations as in Table 1 and Table 2. Statistically significant comparisons (*p* < 0.05) are displayed in bold.

**Table 4:** Net reclassification improvement for adverse cardiovascular events prediction with 5-year SCD risk score categories of European Society and the size of aneurysm plus apical LS-avg.

	Adverse Cardiovascular Events	
	With Events	Without Events
5-year SCD risk score categories of European Society		
<4%, *n*	11	38
≥4%, *n*	14	2
5-year SCD risk score categories of European Society + apical LS-avg		
5-year SCD risk score < 4% + apical LS-avg < −6.6%, *n*	4	34
5-year SCD risk score < 4% + apical LS-avg ≥ −6.6%, *n*	7	4
5-year SCD risk score ≥ 4% + apical LS-avg ≥ −6.6%, *n*	8	0
NRI, %	67.7	
the size of aneurysm		
<20 mm, *n*	11	30
≥20 mm, *n*	14	10
the size of aneurysm + apical LS-avg		
the size of aneurysm < 20 mm + apical LS-avg < −6.6%, *n*	1	29
the size of aneurysm < 20 mm + apical LS-avg ≥ −6.6%, *n*	10	1
the size of aneurysm ≥ 20 mm + apical LS-avg ≥ −6.6%, *n*	5	3
NRI, %	66.2	

The NRI index was used to assess the classification of risk: NRI = [(probability of being correctly upward reclassified/event) − (probability of being incorrectly downward reclassified/event)] + [(probability of being correctly downward reclassified/nonevent) − (probability of being incorrectly classified to an upward category/nonevent)]. NRI: net reclassification improvement; other abbreviations as in Table 1 and Table 2.

## Data Availability

The datasets used and/or analyzed during the current study are available from the corresponding authors upon reasonable request.

## Supplementary Materials

The following supporting information can be downloaded at: https://www.mdpi.com/article/10.3390/diagnostics16040575/s1, Table S1: Inter- and intra-observer variability.

## Abbreviations

HCM	Hypertrophic Cardiomyopathy
SCD	Sudden Cardiac Death
CMR	Cardiac Magnetic Resonance
TTE	Transthoracic Echocardiography
STE	Speckle Tracking Echocardiography
LS-avg	Longitudinal Strain Average
ICD	Implantable Cardioverter Defibrillator
NRI	Net reclassification Improvement
LV	Left Ventricular
NSVT	Non-Sustained Ventricular Arrhythmia
VT/VF	Ventricular Tachycardia/Ventricular Fibrillation
LGE	Late Gadolinium Enhancement
ECGs	Electrocardiograms
MWT	Maximum Wall Thickness
LVEF	Left Ventricular Ejection Fraction
GLS	Global Longitudinal Strain
AHA	American Heart Association
ACC	American College of Cardiology
AMSSM	American Medical Society for Sports Medicine
HRS	Heart Rhythm Society
PACES	Pediatric and Congenital Electrophysiology Society
SCMR	Society for Cardiovascular Magnetic Resonance
ESC	European Society of Cardiology
ANOVA	One-Way Analysis of Variance
RCS	Restricted Cubic Spline

## References

[1] Ommen S.R., Ho C.Y., Asif I.M., Balaji S., Burke M.A., Day S.M., Dearani J.A., Epps K.C., Evanovich L., Ferrari V.A. (2024). 2024 AHA/ACC/AMSSM/HRS/PACES/SCMR Guideline for the Management of Hypertrophic Cardiomyopathy: A Report of the American Heart Association/American College of Cardiology Joint Committee on Clinical Practice Guidelines. Circulation.

[2] Sherrid M.V., Bernard S., Tripathi N., Patel Y., Modi V., Axel L., Talebi S., Ghoshhajra B.B., Sanborn D.Y., Saric M. (2023). Apical Aneurysms and Mid-Left Ventricular Obstruction in Hypertrophic Cardiomyopathy. JACC Cardiovasc. Imaging.

[3] Matsubara K., Nakamura T., Kuribayashi T., Azuma A., Nakagawa M. (2003). Sustained cavity obliteration and apical aneurysm formation in apical hypertrophic cardiomyopathy. J. Am. Coll. Cardiol..

[4] Maron M.S., Rowin E.J., Maron B.J. (2020). Hypertrophic cardiomyopathy with left ventricular apical aneurysm: The newest high-risk phenotype. Eur. Heart J. Cardiovasc. Imaging.

[5] Rowin E.J., Maron B.J., Haas T.S., Garberich R.F., Wang W., Link M.S., Maron M.S. (2017). Hypertrophic Cardiomyopathy with Left Ventricular Apical Aneurysm: Implications for Risk Stratification and Management. J. Am. Coll. Cardiol..

[6] Elliott P.M., Lorenzini M. (2022). Understanding the Prognostic Significance of Left Ventricular Apical Aneurysms in Hypertrophic Cardiomyopathy. JACC Cardiovasc. Imaging.

[7] Maron M.S., Finley J.J., Bos J.M., Hauser T.H., Manning W.J., Haas T.S., Lesser J.R., Udelson J.E., Ackerman M.J., Maron B.J. (2008). Prevalence, clinical significance, and natural history of left ventricular apical aneurysms in hypertrophic cardiomyopathy. Circulation.

[8] Binder J., Jost C.H.A., Klarich K.W., Connolly H.M., Tajik A.J., Scott C.G., Julsrud P.R., Ehrsam J.-E., Bailey K.R., Ommen S.R. (2011). Apical hypertrophic cardiomyopathy: Prevalence and correlates of apical outpouching. J. Am. Soc. Echocardiogr. Off. Publ. Am. Soc. Echocardiogr..

[9] Lorenzini M., Elliott P.M. (2023). Do apical aneurysms predict sudden cardiac death in hypertrophic cardiomyopathy?. Eur. Heart J..

[10] Yang K., Song Y.-Y., Chen X.-Y., Wang J.-X., Li L., Yin G., Zheng Y.-C., Wei M.-D., Lu M.-J., Zhao S.-H. (2020). Apical hypertrophic cardiomyopathy with left ventricular apical aneurysm: Prevalence, cardiac magnetic resonance characteristics, and prognosis. Eur. Heart J. Cardiovasc. Imaging.

[11] Jan M.F., Paterick T.E., Ammar K.A., Khraisat A., Khandheria B.K., Tajik A.J. (2012). Multimodality imaging of left ventricular apical pouch with midventricular cavity obliteration rare variant of hypertrophic cardiomyopathy. J. Am. Coll. Cardiol..

[12] Lee D.Z., Montazeri M., Bataiosu R., Hoss S., Adler A., Nguyen E.T., Rakowski H., Chan R.H. (2022). Clinical Characteristics and Prognostic Importance of Left Ventricular Apical Aneurysms in Hypertrophic Cardiomyopathy. JACC Cardiovasc. Imaging.

[13] Hu X., Bao Y., Zhu Y., Zheng K., Zhang J., Zhou W., Deng Y., Liu Y. (2023). Predicting Left Ventricular Myocardial Fibrosis in Patients with Hypertrophic Cardiomyopathy by Speckle Tracking Automated Functional Imaging. Ultrasound Med. Biol..

[14] Erley J., Genovese D., Tapaskar N., Alvi N., Rashedi N., Besser S.A., Kawaji K., Goyal N., Kelle S., Lang R.M. (2019). Echocardiography and cardiovascular magnetic resonance based evaluation of myocardial strain and relationship with late gadolinium enhancement. J. Cardiovasc. Magn. Reson..

[15] Smiseth O.A., Torp H., Opdahl A., Haugaa K.H., Urheim S. (2016). Myocardial strain imaging: How useful is it in clinical decision making?. Eur. Heart J..

[16] Wang C., Zhou W., Geske J.B., Zhu Y., Tian J., Liu S., Wang H., Chen X., Tang Q., Deng Y. (2024). Clinical Implications of Left Ventricular Apex Mechanics in Patients with Apical Hypertrophic Cardiomyopathy. J. Am. Soc. Echocardiogr. Off. Publ. Am. Soc. Echocardiogr..

[17] Arbelo E., Protonotarios A., Gimeno J.R., Arbustini E., Barriales-Villa R., Basso C., Bezzina C.R., Biagini E., Blom N.A., de Boer R.A. (2023). 2023 ESC Guidelines for the management of cardiomyopathies. Eur. Heart J..

[18] Strachinaru M., Huurman R., Bowen D.J., Schinkel A.F., Hirsch A., Michels M. (2022). Relation Between Early Diastolic Mid-Ventricular Flow and Elastic Forces Indicating Aneurysm Formation in Hypertrophic Cardiomyopathy. J. Am. Soc. Echocardiogr. Off. Publ. Am. Soc. Echocardiogr..

[19] Zeppenfeld K., Tfelt-Hansen J., de Riva M., Winkel B.G., Behr E.R., A Blom N., Charron P., Corrado D., Dagres N., de Chillou C. (2022). 2022 ESC Guidelines for the management of patients with ventricular arrhythmias and the prevention of sudden cardiac death. Eur. Heart J..

[20] Elliott P.M., Anastasakis A., Borger M.A., Borggrefe M., Cecchi F., Charron P., Hagege A.A., Lafont A., Limongelli G., Mahrholdt H. (2014). 2014 ESC Guidelines on diagnosis and management of hypertrophic cardiomyopathy: The Task Force for the Diagnosis and Management of Hypertrophic Cardiomyopathy of the European Society of Cardiology (ESC). Eur. Heart J..

[21] O’Mahony C., Jichi F., Pavlou M., Monserrat L., Anastasakis A., Rapezzi C., Biagini E., Gimeno J.R., Limongelli G., McKenna W.J. (2013). A novel clinical risk prediction model for sudden cardiac death in hypertrophic cardiomyopathy (HCM risk-SCD). Eur. Heart J..

[22] Lang R.M., Badano L.P., Mor-Avi V., Afilalo J., Armstrong A., Ernande L., Flachskampf F.A., Foster E., Goldstein S.A., Kuznetsova T. (2015). Recommendations for cardiac chamber quantification by echocardiography in adults: An update from the American Society of Echocardiography and the European Association of Cardiovascular Imaging. Eur. Heart J. Cardiovasc. Imaging.

[23] Saito M., Okayama H., Yoshii T., Higashi H., Morioka H., Hiasa G., Sumimoto T., Inaba S., Nishimura K., Inoue K. (2012). Clinical significance of global two-dimensional strain as a surrogate parameter of myocardial fibrosis and cardiac events in patients with hypertrophic cardiomyopathy. Eur. Heart J. Cardiovasc. Imaging.

