# Supplementary material for: Incremental Value of Apical Longitudinal Strain in Predicting High-Risk Apical Aneurysms in Patients with Hypertrophic Cardiomyopathy
Source: Diagnostics (Basel). 2026 Feb 14;16(4):575. doi: 10.3390/diagnostics16040575 (PMC12939916; doi:10.3390/diagnostics16040575)
Supplement: Supplementary file 1 [file diagnostics-16-00575-s001.zip › diagnostics-4141762-supplementary.pdf]

Supplementary Table S1 Inter- and intra-observer variability

	Interobserver variability			Intraobserver variability		
	**ICC**	**95% CI**	***p* Value**	**ICC**	**95% CI**	***p* Value**
GLS, %	0.894	0.831-0.934	< 0.001	0.890	0.827-0.932	**< 0.001**
Apical LS-avg, %	0.984	0.975-0.990	< 0.001	0.983	0.973-0.990	**< 0.001**
Mid LS-avg, %	0.966	0.945-0.969	< 0.001	0.964	0.941-0.978	**< 0.001**
Basal LS-avg, %	0.932	0.890-0.958	< 0.001	0.923	0.877-0.952	**< 0.001**

ICC, intraclass correlation coefficient; CI, confidence interval. Statistically significant comparisons ($p < 0.05$) are displayed in bold.
